# Ready, Set, Fuse! The Coronavirus Spike Protein and Acquisition of Fusion Competence

**DOI:** 10.3390/v4040557

**Published:** 2012-04-12

**Authors:** Taylor Heald-Sargent, Tom Gallagher

**Affiliations:** Department of Microbiology and Immunology, Loyola University Medical Center, 2160 South First Avenue, Maywood, IL 60153, USA; Email: theald@lumc.edu

**Keywords:** coronavirus, virus entry, viral pathogenesis, spike protein, carcinoembryonic antigen, angiotensin converting enzyme 2, endocytosis, cathepsin, transmembrane protease, membrane fusion

## Abstract

Coronavirus-cell entry programs involve virus-cell membrane fusions mediated by viral spike (S) proteins. Coronavirus S proteins acquire membrane fusion competence by receptor interactions, proteolysis, and acidification in endosomes. This review describes our current understanding of the S proteins, their interactions with and their responses to these entry triggers. We focus on receptors and proteases in prompting entry and highlight the type II transmembrane serine proteases (TTSPs) known to activate several virus fusion proteins. These and other proteases are essential cofactors permitting coronavirus infection, conceivably being in proximity to cell-surface receptors and thus poised to split entering spike proteins into the fragments that refold to mediate membrane fusion. The review concludes by noting how understanding of coronavirus entry informs antiviral therapies.

## 1. Introduction

Coronaviruses (CoVs) are enveloped RNA viruses causing respiratory and enteric diseases. The CoVs are widely distributed in nature and their zoonotic transmissions into human populations can cause epidemic disease. After entering into respiratory or gastrointestinal tracts, these viruses establish themselves by entering and infecting lumenal macrophages and epithelial cells. The cell entry programs for these viruses are orchestrated by the viral spike (S) proteins that bind cellular receptors and also mediate virus-cell membrane fusions. CoV diversity is reflected in the variable S proteins, which have evolved into forms differing in their receptor interactions and their response to various environmental triggers of virus-cell membrane fusion. As such, seemingly minor differences in CoV S protein structure and function often correlate with striking changes in CoV tropism and virulence. This review focuses on the S: receptor interactions and events leading to membrane fusion and successful cell entry. 

**Figure 1:**
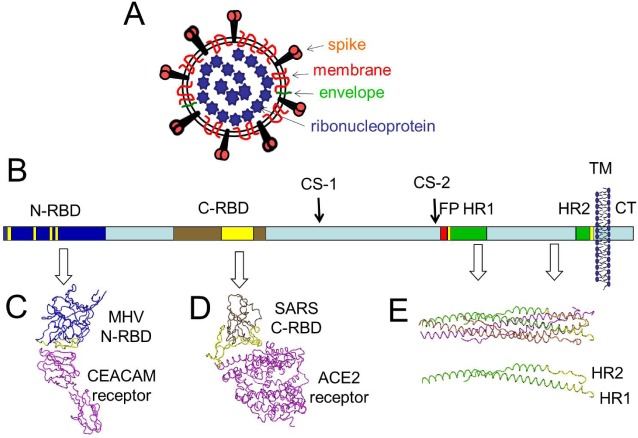
CoV virion and spike protein features. (**A**) Depiction of the virion. The virion core includes a helical ribonucleoprotein core consisting of a ~30 kilobase single-stranded RNA that is surrounded by nucleocapsid (N) proteins. The virion membrane is enriched with viral membrane proteins and includes a small number of envelope proteins. The spikes protrude about 20 nm from the limiting virion membrane; (**B**) Depiction of the S protein. A single S protein is depicted as a rectangle, from N- to C-terminus in left-to-right orientation. Relevant structural features are higlighted as follows: N-terminal receptor binding domain (N-RBD) in dark blue, with receptor binding motif (RBM) in yellow; C-RBD in brown, with RBM in yellow; cleavage sites (CS) 1 and 2, fusion peptide (FP) in red, heptad repeat (HR) regions 1 and 2 in green, with N and C-termini in yellow; transmembrane span ( TM ) depicted as membrane bilayer; cytoplasmic tail (CT) in light blue. The image is adjusted to the primary amino acid scale, which is 1255 residues for the SARS-CoV S protein. *Note that the RBDs that are depicted come from different CoV S proteins; N-RBD from MHV and C-RBD from SARS*; (**C**) Structure of the MHV N-RBD in complex with its CEACAM receptor (PDB 3R4D; reference [8]). The alpha carbon structure of the MHV N-RBD (in blue) is highlighted by RBMs (in yellow). The RBMs contact the N-terminal CEACAM receptor immunoglobulin domain (in purple); (**D**) Structure of the SARS C-RBD in complex with its ACE2 receptor (PDB 2AJF; reference [7]). The SARS C-RBD (in brown) is highlited by RBMs (in yellow). The RBMs contact a virus-binding hotspot [14] on the ACE2 receptor (in purple); (**E**) Structure of the postfusion HR1-HR2 bundle (PBD 1WYY; reference [15]). The HR1-HR2 bundle is depicted as alpha carbon tracings. Each of the three HR1-HR2 are a distinguished by color. To enhance the display, a single HR1-HR2 is extracted from the image and included below the 6-stranded bundle. This HR1-HR2 includes highlights (in yellow) depicting the N-terminal end of the HR1 and the C-terminal end of the HR2. The postfusion configuration is shown as it might appear relative to the fused membrane, when the fusion peptide (not shown) would extend into the fused membrane from the N-terminal end of HR1 and the HR2 would be held into the fused membrane by the TM span.

The S proteins are large; an average CoV S protein contains 1,300 amino acids and 20 asparagine-linked glycans, making the S proteins substantially larger than the glycoproteins mediating receptor binding and membrane fusion of other enveloped viruses. These S monomers are assembled into trimers within the ER of virus-producing cells [1] and incorporated into virus particles at the CoV budding sites, at or near the ER-Golgi Intermediate Compartment [2]. For the human Severe Acute Respiratory Syndrome (SARS) CoV and the Mouse Hepatitis Virus (MHV) CoV, the virion-associated S proteins have been imaged at ~20 angstrom resolution by cryo-electron microscopy [3]. There are about 80 S trimers on each SARS-CoV or MHV particle [4,5]. Each S trimer is held onto viruses by transmembrane anchors located near C-termini, with the vast majority of the S protein mass in extra-virion locations, protruding about 20 nm from the limiting virus membrane of each 80 nm-diameter particle [4] (Figure 1A). 

Atomic–resolution structures are known for domains comprising ~20% of the SARS and MHV S proteins. These include the receptor binding domains (RBDs), which are part of the N-terminal “S1” half of the primary S sequence (Figure 1B). RBD structures in complex with cognate cell receptors (Figure 1C and 1D) have provided several insights into the initial virus-cell interaction [6,7,8,9]. The other atomically-resolved structures amount to a small portion of the C-terminal half of the primary S sequence (Figure 1E) and reveal a coiled-coil made of three alpha helices termed heptad repeat (HR) 1 along with three chains termed HR2 that are bound onto the outer surfaces of the coiled coil (Figure 1E; note the single HR1-HR2 heteromer for clarification). This complex of three HR1s and three HR2s is termed the six-helix bundle (6-HB) and is a very stable, protease-resistant structure that is part of S proteins after they have denatured, or after they have catalyzed membrane fusion (“post-fusion” form) [10,11,12,13]. The structures of HR1 and HR2 in the native or “pre-fusion” S conformations are not known. Furthermore, atomic structures of the remaining ~80% of S proteins are not known for any of the S protein conformations. 

**Figure 2:**
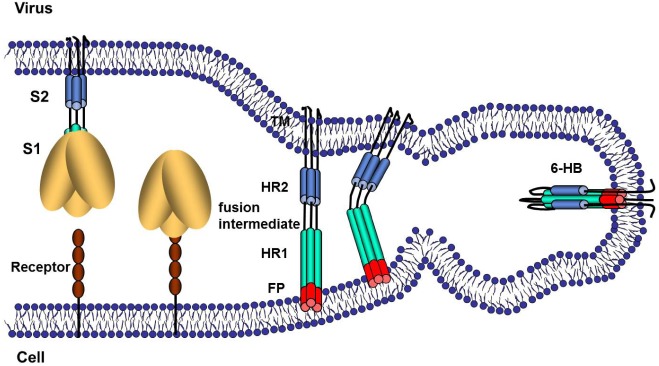
Schematic illustration of CoV S protein-mediated membrane fusion. The illustrations represent several steps of S protein conformational changes that may take place during membrane fusion. In the first step, receptor binding, pH reduction and/or S protein proteolysis induces dissociation of S1 from S2. This step is documented for some MHVs [29,30]. In the second step, the fusion peptide (FP) is intercalated into the host cell membrane. This is the fusion-intermediate stage. In the third stage, the part of the S protein nearest to the virus membrane refolds onto a heptad repeat 1 (HR1) core to form the six-helix bundle (6-HB), which is the final postfusion configuration of the S2 protein.

Models depicting the S-mediated membrane fusion event have extended from knowledge of S protein structures and functions. In part, these models are deemed reasonable because the postfusion 6-HB conformations in SARS and MHV S proteins are so strikingly similar to postfusion forms of influenza HA2, paramyxovirus F2, Ebolavirus GP2 and HIV gp41 [16]. In analogy to these more widely-studied and well-understood viral fusion proteins, the CoV S-mediated membrane fusion process is generally viewed as schematized in Figure 2. Following receptor binding, the CoV S proteins undergo conformational change that exposes hydrophobic domains that likely include a so-called fusion peptide (FP), which embeds into the target cell membrane. Primary sequence data, along with measured fusion activities of site-directed S mutants, suggest that the FPs are near or immediately N-terminal to the HR1 ([17,18]; see Figure 1 for proposed FP location). At this stage in which the FP is in the target cell membrane and the transmembrane (TM) span is in the virion membrane, the S proteins are considered to be in “fusion intermediate” conformations. What follows is the refolding of the regions between the FPs and the TM spans into the postfusion 6-HB configuration, a likely multi-step process that brings opposing membranes into sufficient proximity for lipid mixing and ultimate coalescence. In addition to FP, HR1, HR2 and TM spans, structural elements participating in these later steps include a hydrophobic tryptophan-rich region at the boundary between TM spans and ectodomains [19,20] and a post-translationally palmitoylated region at the boundary between TM spans and cytoplasmic domains [21,22,23]. At the completion of membrane fusion, FPs and TMs are hypothesized to be adjacent to each other, extending from the same end of the 6-HBs and into the membrane bilayer [13,24,25]. In clear analogy to the majority of viruses promoting membrane fusion in this way “HR2” peptides that bind to the HR1 regions of fusion intermediate conformations will potently block CoV cell entry at the membrane fusion stage, most likely by preventing the completion of late-stage refolding into 6-HBs [26,27,28].

This understanding of CoV entry has provided a sophisticated basis for continued exploration of the CoVs. Incentives to continue research on CoV entry are clear–with proven CoV zoonotic potential and extraordinary pathogenicity in humans, a knowledge base of CoV entry offers potential to save lives. There are significant gaps in this knowledge base, and current incentives are to further understand virus-receptor interactions and S protein triggering to catalyze membrane fusion. In adding to the knowledge base, the CoV model system will also bring out new insights of general relevance to virus entry and the membrane fusion. This review covers recent progress in adding to the fund of knowledge on CoV entry.

The sections of this review are organized according to the events of the CoV infection cycle, beginning with the biogenesis and secretion of virus particles. This starting point is chosen to highlight the S protein maturation events in virus-producing cells and to emphasize the continuous preparation for membrane fusion that takes place both in virus-producing and virus-target cells. The subsequent sections proceed to virus-receptor interactions and their roles in inducing S protein conformational changes. Next, the importance of endocytosis is discussed, and S proteolytic scissions at the cell surface and within endosomes are then underscored as critical features of CoV cell entry.

## 2. S protein Morphogenesis and Initial S Protein Proteolysis

The events that ready viruses for cell entry begin in the virus-producing cell. For the CoVs, this beginning stage is in the endoplasmic reticulum of virus-producing cells, where S proteins are synthesized and assembled into trimers. This ER-localized assembly into trimers is a slow process, taking approximately 15 min to generate the greater than 500-kilodalton glycoprotein complexes [1]. Nascent S trimers then transit through the exocytic pathway, embedded by their C-terminal transmembrane domains to vesicle membranes, or to virion membranes if incorporated into viruses at the virus budding sites [2], ultimately residing on the plasma membrane or on secreted virions. It is conceivable that the CoV infection process establishes novel exocytic routes, perhaps with distended organelles able to accommodate secretion of very large S and CoV virion cargo [31]. Exocytic vesicles may even be altered so that the virions and S proteins they house are preserved and properly routed out of the cell. Of note here, the CoV E proteins, small oligomeric transmembrane peptides that accumulate on ER-Golgi intermediate compartment membranes [32] and possess ion channel activities [33], may have a general role in corrupting secretory organelles so that virions are expelled in infectious forms [34]. 

**Figure 3:**
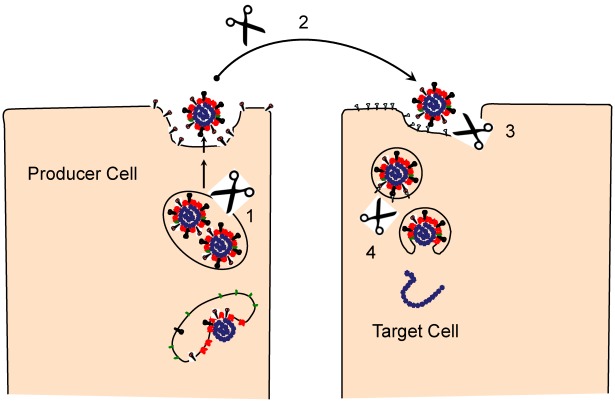
Proteolytic events during the CoV infection cycle. S cleavage events may take place during virus egress from producer cells (1), extracellular transit to target cells (2), on the target cell surface (3), and within the acidified endosome (4).

The virus-producing cell is where the S proteins of some CoVs undergo an early proteolysis (event “1” in Figure 3). The trans-Golgi network houses acid pH-activated serine proteases, notably furin [35], that cleave some S proteins at positions immediately C-terminal to multibasic cleavage sites, at a place that we labeled as cleavage site 1 (CS-1) in Figure 1. Scission at CS-1, also designated as S1-S2 cleavage, separates the RBDs and fusion machineries into noncovalently-associated peripheral S1and transmembrane-anchored S2 fragments. That furin proprotein convertases are the responsible proteases has been established by using peptide furin inhibitors, which arrest the S cleavages [36]. Notably, only a subset of CoVs have S proteins with the multibasic cleavage sites, and of these, only a select few have the consensus K/R-X-K/R-K/R motif at the P4-P1 position. Thus, in most CoV infections, S protein populations are left intact or are only partially cleaved in virus-producing cells. For those S proteins that do harbor the cleavage site motifs, site-directed mutation of the multibasic residues to render “uncleavable” states does not destroy virus infectivity [37,38]. Experiments involving pharmacologic inhibition of furin complement these evaluations of mutant viruses, and reveal that reduced furin activities greatly reduce S cleavages without diminishing virus infectivities [36].

S1-S2 cleavage does, however, increase the potency of CoV S proteins to mediate cell-cell fusions [39,40]. This influence on syncytial developments may explain some of the evolutionary diversity of S1-S2 cleavages. Syncytia may benefit CoVs in certain niches, *i.e.*, during acute infections, by promoting rapid virus dissemination, and this proviral effect may select for the multibasic cleavage motifs. In other niches, *i.e.*, during chronic or smoldering infections, weakened syncytial cells may have greatly compromised virus-producing capacities, and this antiviral effect may select against the same multibasic peptide sequences. Additional, entirely distinct selective forces may target the basic peptide motifs, as the K/R-X-K/R-K/R stretch can mediate virus binding to heparan sulfate, a known receptor for murine CoVs [41]. Again, adsorption to heparan sulfate can be proviral in tissue culture and antiviral *in vivo*, making for complex selective forces impinging on multibasic S peptide motifs. 

Exactly how S1-S2 cleavages promote syncytia (cell-cell fusion), often without significant effects on virus entry (virus-cell fusion), are not yet entirely clear. Notably, the S1-S2 cleavage site is distant from any hydrophobic peptides, hundreds of residues from the presumed fusion peptide (Figure 1). This positioning is clearly unlike the analogous cleavage sites on influenza hemagglutinin and HIV gp160, which are adjacent to fusion peptides and thus create hydrophobic N-termini for target membrane insertion. With simple models of S1-S2 cleavage liberating an N-terminal fusion peptide appearing unsatisfactory, there have been hypotheses for additional S cleavages nearer to the fusion peptides. Support for this hypothesis that S is a pre-pro-protein is now in place. As these cleavages to the final fusion-active S likely take place at later times in the continuum of virus assembly, secretion and entry into new cells, they are described in detail in the following sections.

## 3. Engaging the CoV Receptors

All CoVs, regardless of their pre-primed S protein cleavage status, initiate new infections by binding receptors on virus-target cells. These receptor-binding events, their variabilities amongst the CoVs, and their potential for priming subsequent virus-target cell membrane fusions, are discussed here. 

A fascinating feature of the CoVs is in the diversity of receptor-binding events. This diversity shows up readily upon inspection of the CoV receptors, which include integral-membrane proteins and sugars that, as a group, have no obvious structural similarities (Table 1). This variation presents interesting questions regarding which receptors are simply mediating CoV adsorption and which might be doing more; *i.e.*, generating fusion-promoting S protein conformational changes and/or directing viruses through particular endocytic routes. Several studies have demonstrated that CoV receptors may not require special endocytic functions beyond S binding: Integral-membrane protein receptors lacking cytoplasmic tails and thus unlikely to regulate virus endocytosis or transduce virus-promoting signals will still provide for virus entry [42,43]. In the case of MHV-CoV and its CEACAM receptors (Table 1), cell entry can be catalyzed by soluble, exogenously-added CEACAM ectodomains [44,45,46], indicating that this receptor can advance entry through S binding, and that a role for the receptor in guiding bound viruses into particular endocytic organelles is not absolutely essential. 

Diversity of receptor interaction is also readily evident by examining the CoV S proteins. The S proteins are striking in that, as a group, they possess at least two RBDs. One RBD encompasses the S1 N-terminal domain (N-terminal RBD in Figure 1 and Table 1). Recent structural resolution of this NTD from MHV-CoV has revealed a galectin-like beta-sandwich fold, and indeed, the NTDs from related CoV strains (human CoV-OC43 and bovine CoV) are functional lectins and do bind various acetylated sialic acids [8]. Remarkably, the resolved MHV NTD structure lacks a peptide loop necessary for sugar binding, and thus does not operate as a lectin, instead possessing high affinity for the CEACAM proteins. The intriguing indications here are that the N-terminal RBD structures can evolve disparate sugar or protein receptor-binding activities. The findings are valuable in considering CoV receptor switching as it relates to host range and zoonotic infection potential. 

**Table 1:** Coronaviruses and their receptors. Viruses include TGEV: Transmissible Gastroenteritis Virus, PRCoV: Porcine Respiratory Coronavirus, HCoV-229E: Human Coronavirus 229E, HCoV-NL63: Human Coronavirus Netherlands-63, SARS-CoV: Severe Acute Respiratory Syndrome-related Coronavirus, MHV: Murine Hepatitis Virus, HCoV-OC43: Human Coronavirus OC-43, BCoV: Bovine Coronavirus. Cell receptors for N-terminal virus RBDs include sugars and CEACAM1: Carcinoembryonic Antigen-related Cell Adhesion Molecule 1. Cell receptors for C-terminal RBDs include APN: Aminopeptidase-N, and ACE2: Angiotensin Converting Enzyme 2.

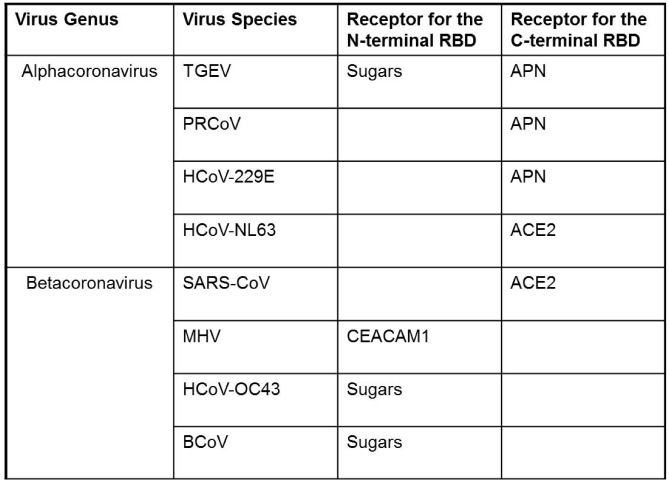

The other CoV RBD encompasses a region near the middle of S1 and has been termed the C-terminal domain (CTD). The C-terminal RBD structures of human SARS (shown in Figure 1D) and NL63 (not shown) CoVs are completely distinct, a beta sheet architecture in SARS and a beta sandwich in NL63, yet these two CTD RBDs bind to the same region within the ACE2 receptor, termed the “virus binding hotspot” [6,8,14,47]. In the SARS S and CTD, there are well-described mutations that change affinities for human, civet cat and bat ACE2 receptors [9,47,48], and as expected, virus affinity for the human ACE2 correlates with human infection and epidemic human to human transmission [47]. The CTD structures on those CoVs that bind APN (or other as yet undetermined receptors) are not yet available, but once determined, may foster analogous identification of S mutations that change affinities for far more diverse receptors. Such studies will promote understanding of how CoV C-terminal RBDs, like the N-terminal RBDs, possess flexible architectures that can evolve disparate receptor-binding activities, again contributing to acquisition of new host ranges and zoonoses. 

Notably, the existence of two CoV RBDs can make one dispensable. The MHVs, having CEACAM-binding sites on NTDs, readily delete CTD segments during passage in tissue culture [49,50]. These deletion-mutations have profound influences on CoV virulence, greatly decreasing the murine virus neurovirulence [51]. Another interesting example is with the porcine CoVs. Transmissible gastroenteritis virus (TGEV), with two functional RBDs (Table 1), can infect both respiratory and enteric tracts [52]. However, naturally-occurring mutants with substitutions or deletions in N-terminal RBDs lack lectin activities [53] and are strictly limited to the respiratory tract and therefore are attenuated [54,55]. Thus a dual RBD architecture may correlate with *in vivo* tropism and virulence, likely due to a complexity of multiple receptor interactions that the CoVs employ in the natural animal environment. 

Diverse receptor interactions precede the CoV S-mediated fusion reactions, and while the binding of relatively low-affinity carbohydrate receptors may not generate fusion-promoting S protein conformations protein receptors that bind S proteins at high affinity clearly do, as evidenced most extensively by studies with MHV. Early seminal findings using MHV demonstrated that alkaline pH increased S fusion activities and S1 release, a readily observed conformational change [29]. Soluble CEACAM receptors were then found to catalyze S1 release [30,56], and biological relevance was subsequently established when soluble receptors were found to support infectious MHV entry into CEACAM-negative cells [44]. More recently, using an MHV2 strain, soluble CEACAM receptors generated SDS-resistant S protein trimers with unique lipophilicities and protease susceptibilities [57]. Thus the MHV model system divulges relatively stable CEACAM receptor-induced S conformations that are quite likely the intermediate structures on the way to membrane fusion (see Figure 2 for hypothetical illustration of receptor-induced generation of fusion intermediate S structures). 

What is not disclosed by the MHV model system, however, is how CEACAM binding to the NTD RBDs can uncover the fusion machinery in S2. In the primary S sequence, the NTD RBDs are distant from the fusion-inducing peptides. Structural biologists will undoubtedly address this issue most effectively, but at present, intriguing molecular genetic data strongly suggest connections between RBDs and fusion apparati in the context of the native S trimers. One of the first findings in support of such connections was with the identification of a mutation in the fusion domain that destroyed an antibody epitope in the RBD [58]. There have been numerous comparable observations since then. Indeed, MHV evolution, both *in vitro* and *in vivo*, frequently fixes substitution mutations in or near the RBDs, and into the fusion regions [59,60,61]. In several instances, these mutations do not change RBD affinities for CEACAMs, but do increase or decrease the tendency for S proteins to undergo CEACAM-induced transitions into fusion-intermediate forms [57,62], suggesting that one selective pressure relates to MHV responsiveness to the CEACAM receptor “trigger”. Finally, the fact that these mutations are frequently associated with reduced virus virulence creates the additional suggestion that any complete understanding of CoV disease requires appreciation of how receptors operate as fusion triggers. 

Relative to CEACAM and its effect on MHVs, the roles of ACE2 and APN receptors in triggering structural transitions of human CoVs is less clear. It is, for example, uncertain whether ACE2 binding to SARS or NL63 S proteins dissociates RBDs from fusion modules and exposes hydrophobic “fusion peptides”. Compelling data do, however, suggest that ACE2-bound S proteins are set up for proteolytic scissions that activate membrane fusion, as described in the following sections. The human CoV receptors, therefore, may be similar to the murine CEACAM CoV receptors in that their binding energies drive S proteins into intermediate forms with exposed proteolytic substrate sites. Host proteases may then ultimately liberate the fusion machineries and allow them to operate in CoV entry.

Finally, there is the question of whether virus entry and the receptor-induced conformational changes leading to membrane fusion take place at the plasma membrane or at a later time, after viruses have entered into endosomes. This question is explored in the following section.

## 4. Endocytosis Following Coronavirus: Cell Binding

Endocytosis is the principal cell entry route for most viruses [63]. Receptor-mediated endocytosis is an advantageous entry route as it can allow the virus (in endosomes) to pass through a barrier of cortical actin and traffic deeply into the cell before fusing and expelling its genome to cytosol [64]. The endosome also bathes the virus in an acidic, proteolytic environment that is frequently necessary to activate viral fusion. It appears that the majority of CoVs enter cells via endocytosis. However, a subset of CoVs have been thought to enter in a more straightforward direct fusion with the plasma membrane, making the CoVs useful for dissecting various cell entry routes.

The idea that some CoVs do not endocytose but rather enter by fusion directly at the plasma membrane comes from several findings, including the fact that several CoVs readily induce syncytia, *i.e.*, plasma membrane-localized cell-cell fusions. In those studies where pH effects were investigated, syncytial developments increased in slightly alkaline media [56], suggesting no need for endosomal acid exposures to activate fusions. Similarly, some CoVs, notably the JHM strain of MHV, are impervious to inhibitors of endosomal acidification [65]. Finally, when incubated with receptor-bearing cells at extraordinarily high input multiplicities, some CoVs can induce immediate cell-cell fusions, often called “fusion-from-without” [66]. But none of these observations necessarily confirm that CoVs naturally enter cells via fusion at the plasma membrane. First, it may be inappropriate to equate syncytial formation (cell-cell fusion) with virus entry (virus-cell fusion), because these two processes have distinct requirements, as revealed by S mutations that abolish syncytial formation without affecting virus infectivity, as discussed later in this review. Second, increased syncytial activity at basic pH may reflect the pH optima of the cell-surface trypsin-like proteases that create fusion-active S fragments, and may primarily apply to fusions mediated by S proteins that are not associated with virions. Third, the resistance of some CoVs to endosome neutralization can indicate fusion in early non-acidic endosomes, but cannot be used to conclude that virus-cell fusion takes place at the plasma membrane. Indeed there are several reports, particularly in HIV research, that viruses acquiring membrane fusion competence at neutral pH may still utilize endosomal networks during entry [67]. This leaves the “fusion from without” phenomena, clearly plasma membrane-based virus-cell fusion events, but perhaps occurring with very low frequencies and thus only observed in artificial experimental conditions in which viruses are applied to cells at extremely high doses. 

That the CoVs generally utilize cellular endocytic machinery during entry is also supported by studies aimed at dissecting CoV entry pathways [68]. Many of these investigations assess the efficiencies of CoV infections after specifically arresting clathrin, or caveolar, or cholesterol-dependent endocytosis with pharmacologic agents and overexpressed dominant-negative proteins. Valuable results have indicated various CoV entry pathways; caveolar endocytosis for human 229E-CoV [69], clathrin-mediated endocytosis for SARS-CoV [42], cholesterol-raft entry for MHV [70], clathrin and caveolar–independent endocytosis for feline CoV [71]. However, complete blockade of CoV entry is rarely achieved by suppressing any single endocytic pathway, suggesting that multiple cell entry routes can be utilized by the CoVs. That CoVs can resourcefully invade cells in alternate routes is exemplified by successful SARS-CoV entry when both clathrin and caveolar endocytosis is blocked [72]. Similarly, SARS-CoV entry, an ACE2 receptor-mediated process that typically traffics viruses into acidic organelles [69], is entirely distinct when mediated by antibodies and Fc-gamma-receptor II. In this so-called antibody-dependent enhanced entry, infection is entirely unaffected by the endosome -neutralizing agents and protease inhibitors that arrest the ACE2-mediated entry process [73]. In summary, different CoV endocytic entry routes can culminate in successful infections. 

In the endosomes, CoVs are bathed in an acidic and often proteolytic environment. The S proteins from the majority of CoV strains clearly require acid exposures to acquire membrane fusion competence. However, it is unclear whether protons are the endosomal factor(s) triggering fusion per se, as acidification itself does not enhance S-mediated fusions [29]. Acid pH-activated endosomal proteases may be the more likely triggers [74,75,76]. Hence an interesting question arises as to whether entry depends on acidic endosomal conditions solely because the fusion-activating protease(s) are themselves acid pH-dependent. This is a question considered in studies of filovirus entry [77] and the CoVs present an additional study model here because the effects of protonation on CoV S fusion structures can be dynamically evaluated by magnetic resonance [78]. The CoV model system appears poised to contribute to a general understanding of proton-triggers of virus entry. 

## 5. Virus Entry, S Proteolysis and Membrane Fusion

The initial and most obvious S proteolysis takes place in virus-producing cells, at the “CS-1” position (Figure 1), to generate secreted viruses with peripheral (S1) and transmembrane (S2) fragments (event “1” in Figure 3). As stated in the previous section on S morphogenesis, these S1-S2 scissions are not observed in all CoVs. For those CoVs with uncleaved S proteins, the relevant scissions take place during virus entry (events “3” and “4” in Figure 3). Simmons *et al*. [76] and Matsuyama *et al*. [79] made the initial seminal discovery that cathepsin L inhibitors block SARS S mediated virus entry and that trypsin proteolysis bypasses this inhibition, making it clear that cell entry via SARS S proteins depends on a proteolytic cleavage by cathepsin L. These were enlightening results that arose nearly concurrently with the discovery that Ebolavirus entry requires cathepsin cleavage of its fusion glycoproteins [80], thus establishing that endosomal proteases can promote the entry of different enveloped viruses. Subsequent questions regarding the S cleavages were addressed biochemically, and it was found that SARS S ectodomains were cleaved by cathepsin L at the S1-S2 position (CS1 in Figure 1) [81]. This made it appear that, depending on the CoV strain, S cleavage at CS1 either takes place early in the infection cycle, by furin-like serine proteases in the trans-Golgi network of virus-producing cells, or later in the cycle, by cathepsin L cysteine proteases in the endosomes of virus-target cells, with similar ultimate outcomes. Supporting this view, precleaving the SARS S by inserting a consensus furin cleavage site 1 freed the virus from its dependence on endosomal acidification [82]. More recent findings, however, have added complexity to these S proteolytic cleavages. An additional mutibasic peptide motif (at a position we designated in Figure 1 as “CS2”) was recognized in infectious bronchitis CoV (IBV) S, and shown via mutagenesis to be essential for IBV cell entry [83]. The homologous position in SARS S was also found to be highly relevant, as its mutation into a consensus furin cleavage motif allowed for SARS S–mediated entry without the need for exogenous trypsin proteases [84]. This second cleavage site (CS2) has since been biochemically confirmed via identification of the shorter C-terminal S fragment, both with SARS S proteins [85] and with MHV S proteins [57]. Although research on S proteolytic cascades is ongoing, the present findings are consistent with a model in which S proteins are cleaved multiply, likely first at the CS1 (S1-S2) position, and then at CS2 positions adjacent to the FP, to generate fusion intermediate structures of the type depicted in Figure 2. The CS2 proteolytic removal of sequences that are N-terminal to the FP may permit the rapid completion of the S refolding events that generate membrane fusion and the 6-HB configuration.

There is also the question of precisely when the S proteins are available for proteolysis. The weight of current evidence argues that the target cell, not the free extracellular space, is the site of S proteolysis. For example, cell-free SARS-CoV virions were rendered non-infectious by trypsin pre-treatment of free virions [72]. However, the same trypsin exposures enhanced infectivities of cell-bound viruses [76,79] (event “3” in Figure 3). These findings suggest that it is the cell receptor-associated S proteins that are in unique protease-responsive structural forms. While these experiments used a protease that is not encountered by incoming respiratory CoVs such as SARS-CoV, the findings still do imply that S conformations change significantly upon receptor interaction or endocytosis, and in turn illuminate the importance of S conformation and subcellular location to the effects of S proteolysis. 

That exogenously added trypsin could increase the infectivities of cell receptor-associated SARS-CoV suggests that cell-bound ectoprotease(s) might be the natural cofactors for CoV entry. Indeed, CoV S can be primed for fusion by recently described cell-surface proteases known as Type II Transmembrane Serine Proteases (TTSPs) [85,86,87,88,89]. The TTSPs may reside in plasma membrane microdomains, although this possibility is not yet validated, and they may be available to incoming viruses at the receptor-interaction stage, to cleave at the CS1 and/or CS2 positions. 

The prototype TTSP, enteropeptidase, was discovered nearly a century ago, and the most recent decades of research have revealed at least 20 additional family members [90,91]. Family member variations are in the domains between C-terminal protease and N-terminal transmembrane anchors. The roles of these domains in viral glycoprotein cleavage, if any, are unknown, but they may dictate substrate specificities by influencing protease subcellular localization or association with other membrane proteins [90]. Only a subset of TTSPs have been evaluated as mediators of virus entry. Principal amongst these evaluated TTSPs is transmembrane protease, serine 2 (TMPRSS2). First described in 1997 [92], a role for TMPRSS2 in normal physiology remains elusive, largely because TMPRSS2 knockout mice develop normally [93]. But TMPRSS2 may have a fundamental organism-wide role, as it is expressed in tissues such as the heart, liver, prostate, intestines, and most notably, the lung [92]. Abundance in the lung has prompted screening of respiratory viruses for TMPRSS2 activation of their glycoproteins. TMPRSS2 can cleave the hemagglutinins (HAs) of influenza viruses, leading to their increased replication, including the highly pathogenic 1918 strain [94,95]. TMPRSS2 also cleaves the membrane glycoprotein of SARS-CoV. Matsuyama *et al*. discovered that increased levels of TMPRSS2 resulted in more cell death and increased SARS-CoV viral replication in vitro [86]. Shulla *et al*. confirmed and expanded this finding by noting that TMPRSS2 specifically increases SARS-CoV entry using pseudotyped reporter assays [88]. TMPRSS2 cleavage may also contribute to immune evasion. By cleaving S from infected-cell surfaces, TMPRSS2 releases S fragments that bind and incapacitate neutralizing antibodies [87]. Hence the *in vivo* infection process may be heavily influenced by TMPRSS2 and related family members, both at virus entry and release, influencing pathogenesis and immune response. 

Another TTSP, Human Airway Trypsin-like Protease (HAT or TMPRSS11d), has brought out enlightening details concerning member-specific proteolytic properties. In the context of influenza HA cleavage, HAT has a broader cleavage capacity than TMPRSS2, proteolyzing HA both in virus-producing cells and in progeny viruses bound to target cell receptors [96]. Thus HAT, not TMPRSS2, is the more relevant protease operating on influenza at the virus entry stage. In the context of SARS-CoV and S cleavage, HAT again exhibits a broader cleavage capacity than TMPRSS2, making it so that HAT can cleave and enhance S-mediated virus entry either in virus-producing cells or on the surface of virus-target cells [89]. However, overexpressed TMPRSS2 bypasses the requirement for endosomal acidification and therefore cathepsin activation [86,88], but HAT does not similarly replace cathepsins in SARS-CoV entry [89]. Thus a further dissection of the various TTSP substrate specificities will be necessary to precisely identify those most relevant to virus infection, and efforts in this regard are continuing. For example, the first paper to examine TTSPs in the context of SARS entry found that TMPRSS11a was capable of slightly enhancing SARS S bearing pseudoparticles [85]. Subsequent findings indicated that, while TMPRSS11a was capable of modestly increasing SARS entry at low levels of the protease, TMPRSS2 was a more potent activator of entry [88]. Most recently, various TTSPs including TMPRSS3, TMPRSS4, TMPRSS6, and Hepsin, have been evaluated, yet none have exceeded TMPRSS2 in augmenting SARS-CoV entry [87,89]. Other candidate TTSPs worth testing in SARS-CoV entry assays are MSPL and TMPRSS13, as they have been found to cleave certain influenza HAs [97]. 

While the TTSPs may be the most relevant proteases in natural CoV infections, they are clearly dispensable in several tissue culture settings. This is because cathepsins, specifically cathepsin L, will proteolytically activate SARS CoV S proteins following virus endocytosis (event “4” in Figure 3) Multiple proteases with possibly redundant virus entry functions make it difficult to discern which proteases are necessary for viral entry. This difficulty is perhaps most recognized by the fact that the presumed proteolytic activation of another human CoV, NL63, is entirely unclear. NL63 S-mediated entry was not affected by preventing endosomal acidification or by cathepsin inhibitors [98]. While NL63 is similar to SARS in that it binds to the same receptor, ACE2, the protease responsible for NL63 S cleavage remains a mystery.

## 6. Therapeutics and Inhibition of CoV Entry

Currently, vaccination is the best clinical approach to eradicating viruses. Experimental SARS-CoV vaccine immunogens include inactivated whole viruses, entire S trimers, and RBDs (reviewed in [99]). All formulations elicit protective antibodies that prevent virus entry, some protecting against lethal SARS-CoV challenge, with most antibodies operating sterically to block virus-receptor engagement [100,101]. However, protective antibodies can operate variably and in novel ways, as shown by a human monoclonal antibody directed against the trypsin cleavage site in S that reduced viral titers and pathology associated with SARS-CoV infection [102]. Interestingly, despite binding to a proteolytic substrate site, this antibody did not prevent S cleavage but rather prevented membrane fusion by an unknown mechanism [102]. The antibody was created in transgenic mice that produce human immunoglobulins, representing an interesting new production method for passive human immunization. These new approaches signal subunit vaccination and therapeutic antibodies as attractive future clinical options, but the methods must be evaluated cautiously, as anti-SARS antibodies can permit virus entry via Fcγ receptors, bypassing the ACE2-dependent entry route and actually enhancing disease [73]. 

Attractive natural-product anti-CoV agents include lectins that bind avidly to the unprocessed, high-mannose carbohydrates on CoV S proteins. One such lectin is griffithsin, an oligomer with six high-affinity mannose binding sites. Griffithsin is known to block HIV infection via envelope protein binding [103]. Griffithsin was found to have analogous anti-SARS activity, preventing SARS-CoV infection and pathology both *in vitro* and in a mouse disease model, without incidental cytotoxity [104]. Other potential anti-human CoV lectins include the mannose-binding lectin, which arrests SARS-CoV entry [105] and the plant lectins *Galanthus nivalis* agglutinin, *Hippeastrum* hybrid agglutinin, and *Urtica dioica* agglutinin, which, to date, have only been evaluated for anti-MHV activity [106]. Notably, most of these lectins do not affect binding of S to the receptor, but rather seem to inhibit entry at a later stage [105,106]. 

Several of the proteases described in this paper are potential antiviral therapeutic targets Indeed, furin-inhibiting peptides arrest cell-cell fusions mediated by coronaviruses MHV and IBV (infectious bronchitis virus) [36,83]. A novel druglike cathepsin L inhibitor, identified by high-throughput screening, prevents Ebola and SARS-CoV cell entry [107]. TTSPs also promise to be antiviral drug targets. With their proteolytically active sites exposed to the extracellular milieu, hydrophilic inhibitors that remain excluded from cells may be suitable, thus reducing medicinal chemistry and toxicity problems. Transient, pharmacologic TMPRSS2 suppression may have no health consequences, given that the TMPRSS2-null mouse is phenotypically normal. Targeting other cellular proteases, such as furins or cathepsins, may have unintended or toxic side effects due to their importance in normal physiology. Finally, targeting host proteins is a sensible adjunct to the design of inhibitors targeted toward highly-mutable virus protein targets. 

In addition to preventing CoV entry, inhibiting TTSPs and other proteases would also prevent the maturation of other viral glycoproteins, blocking entry of a variety of viruses. An example of a potentially multi or pan-viral protease inhibitor is the human cathepsin L inihibitor tetrahydroquinoline oxocarbazate that blocks both SARS and Ebola glycoprotein mediated entry *in vitro* [107]. While animal studies have yet to be published on that cathepsin inhibitor, Bahgat et al. recently reported that a cocktail of serine protease inhibitors reduced influenza infection and subsequent disease in mice [108]. Additionally, more historic reports used single agent serine protease inhibitors such as camostat, leupeptin and aprotinin to decrease influenza infection both in mice and in humans [109,110]. Zihnov *et al*. reported that aerosol delivery of aprotinin shortened influenza symptomatology in humans [110]. It is possible that the antiviral aprotinin target was TMPRSS2, given that TMPRSS2 is abundant in lungs and that aprotinin cannot penetrate the plasma membrane. In support of this possibility, TMPRSS2 knockdown with peptide-conjugated morpholino oligomers, which are stable antisense oligonucleotides, rendered TMPRSS2 positive human airway cells resistant to influenza virus replication [111].

In the end, inhibiting human CoVs may require a multi-target approach. Blocking multiple proteases along the entry pathway might be combined with lectins, and also with peptide-based fusion inhibitors. Fusion of the viral membrane with the target cell membrane can be arrested by peptides mimicking the S heptad repeat region 2 (HR2), which bind and inactivate fusion intermediate S forms [26], as mentioned earlier in this review. Different HR2 peptides have been investigated with IC50s in the nanomolar to micromolar range [26,112]; these high affinities and specificities are similar to the HIV HR2 peptide, enfuvirtide, currently used in humans [113]. Further refinement of the HR2 peptides, and development of peptidomimetics, might enhance antiviral activities and make them even more suitable for anti-CoV treatment [27]. HR2 peptides cover a broad viral target and thus impose barriers against virus mutational escape routes. Mutations in HR1 that both confer HR2 resistance and preserve virus viability are rare [114]. While HR2 peptides could be extremely potent entry inhibitors, our understanding of SARS entry makes multiple therapeutics targeting viral binding and fusion feasible and most likely of great value. Certainly, a multifaceted approach to SARS entry inhibition would be the most likely to succeed. 

## 7. Future Directions

The CoV S peplomers directing cell entry are the principal viral components communicating with diverse extracellular environments. These arrays of extracellular selective pressures fix diversity into the genetically-plastic CoV S proteins. Novel S structures allow the CoVs to expand into different ecological niches; zoonotic emergence of the SARS-CoV being the prime example of this emergence and its consequences. Understanding S variation, structure, and entry mechanisms are therefore central to combating potential epidemic CoV transmissions.

The natural diversity of the CoV S proteins provides insights into evolution and mechanism of cell entry. For example, amongst the CoVs, there are central distinctions in RBDs and fusion–activating triggers. These differences can dramatically influence pathogenicity by redirecting cell tropism, viral entry routes and rates. The CoVs are facile models to correlate variations in cell entry with disease potential. 

The CoVs are also excellent tools to reveal new host cell components facilitating entry. Of relevance to disease are the possible species and organ-specific variations in CoV receptors and S-activating proteases. One of the next key steps in CoV research will be to move toward *in vivo* dissection of entry mediators and relate disease manifestations to their presence and abundance in distinct tissues and species.

Increasingly sophisticated *in vivo* CoV models will also be useful in evaluating new antiviral strategies. Notably, the infection models and the prototype antiviral agents promise to deliver therapies that will both guard against potentially serious zoonotic CoVs and reveal more broad–spectrum antivirals. For example, TTSP inhibitors may be suitable therapeutics for a variety of respiratory viruses that rely on proteolysis for cell entry. CoV research will continue to reveal features of virus operating mechanisms and clinically applicable antiviral strategies for the foreseeable future.
